# Obtaining Valid Laboratory Data in Clinical Trials Conducted in Resource Diverse Settings: Lessons Learned from a Microbicide Phase III Clinical Trial

**DOI:** 10.1371/journal.pone.0013592

**Published:** 2010-10-27

**Authors:** Tania Crucitti, Katrien Fransen, Rashika Maharaj, Tom Tenywa, Marguerite Massinga Loembé, Kailapuri Gangatharan Murugavel, Kevin Mendonca, Said Abdellati, Greet Beelaert, Lut Van Damme

**Affiliations:** 1 HIV/STI Reference Laboratory, Institute of Tropical Medicine, Antwerp, Belgium; 2 HIV Prevention Research Unit, Medical Research Council, Durban, South Africa; 3 STI Reference Laboratory, Makarere University, Kampala, Uganda; 4 Département de Médecine Sociale et Préventive, Université Laval, Quebec, Canada; 5 Unité de Recherche en Santé des Populations, Centre Hospitalier Affilié Universitaire de Québec, Quebec, Canada; 6 Centre Hospitalier Affilié Universitaire de Québec, Quebec, Canada; 7 Y. R. Gaitonde Center for AIDS Research and Education (Y. R. G. CARE), Voluntary Health Services Campus, Chennai, India; 8 Population Health and Clinical Research, St. John's Medical College, Bangalore, India; 9 CONRAD, Arlington, Virginia, United States of America; 10 Family Health International, Research Triangle Park, North Carolina, United States of America; Tulane University, United States of America

## Abstract

**Background:**

Over the last decade several phase III microbicides trials have been conducted in developing countries. However, laboratories in resource constrained settings do not always have the experience, infrastructure, and the capacity to deliver laboratory data meeting the high standards of clinical trials. This paper describes the design and outcomes of a laboratory quality assurance program which was implemented during a phase III clinical trial evaluating the efficacy of the candidate microbicide Cellulose Sulfate 6% (CS) [1].

**Methodology:**

In order to assess the effectiveness of CS for HIV and STI prevention, a phase III clinical trial was conducted in 5 sites: 3 in Africa and 2 in India. The trial sponsor identified an International Central Reference Laboratory (ICRL), responsible for the design and management of a quality assurance program, which would guarantee the reliability of laboratory data. The ICRL provided advice on the tests, assessed local laboratories, organized trainings, conducted supervision visits, performed re-tests, and prepared control panels. Local laboratories were provided with control panels for HIV rapid tests and *Chlamydia trachomatis*/*Neisseria gonorrhoeae* (CT/NG) amplification technique. Aliquots from respective control panels were tested by local laboratories and were compared with results obtained at the ICRL.

**Results:**

Overall, good results were observed. However, discordances between the ICRL and site laboratories were identified for HIV and CT/NG results. One particular site experienced difficulties with HIV rapid testing shortly after study initiation. At all sites, DNA contamination was identified as a cause of invalid CT/NG results. Both problems were timely detected and solved. Through immediate feedback, guidance and repeated training of laboratory staff, additional inaccuracies were prevented.

**Conclusions:**

Quality control guidelines when applied in field laboratories ensured the reliability and validity of final study data. It is essential that sponsors provide adequate resources for implementation of such comprehensive technical assessment and monitoring systems.

**Trial Registration:**

ClinicalTrials.gov NCT00153777 and Current Controlled Trials ISRCTN95638385

## Introduction

Microbicides are female initiated methods for the prevention of HIV and other sexually transmitted infections (STIs). Several phase III clinical trials assessing the effectiveness of microbicides have been conducted over the last decade. Trials were mostly implemented in developing countries, where the need for microbicides is the highest in view of the relatively high HIV incidence rates among women.

Large scale multi-centre phase III clinical trials often face challenges in terms of adequate and appropriate clinical study facilities, equipment and staff. This may especially be true for the local laboratories delivering services for trials, which have to ensure delivery of accurate and reliable data. Laboratories operating in resource constrained settings, often lack experience with multi-site clinical trials conducted under an Investigational New Drug application, and have difficulties implementing Good Clinical Practices.

The phase III clinical trial evaluating the effectiveness of Cellulose Sulfate (CS) for HIV and STI prevention was conducted in five study sites over two continents [1]. Study sites differed considerably in terms of clinical trial experience, laboratory testing experience and even availability of laboratory infrastructures. Only one study site in Kampala, Uganda, had all testing done within the same facility. The other four study sites used two or three different laboratories and the transportation of specimens between each of them was an additional challenge. Laboratory infrastructures varied significantly between the study sites, and in some sites renovations had to be conducted before the study could be set up.

Although English was the study language, Standard Operating Procedures (SOPs) and other laboratory documents had to be translated; communication with staff was done in French for the Benin laboratory staff. In Mudhol, India, laboratory staff had difficulties in reading and understanding English. Therefore additional trainings in Kannada (the local language) had to be organized by the local senior staff.

In this publication we present the outcome of activities undertaken to guarantee the quality of the laboratory data in the Phase III clinical trial of CS.

## Materials and Methods

The study was a randomised, double-blind and placebo-controlled trial of cellulose sulfate formulated as a vaginal gel and was conducted in three African and two Indian sites. The study was sponsored by CONRAD who selected the HIV and Sexually Transmitted Infections Reference Laboratory from the Institute of Tropical Medicine, Antwerp, Belgium, as the international central reference laboratory (ICRL) for the study. The study was approved by the Institutional Review Board of the Eastern Virginia Medical School and of the Institute of Tropical Medicine; and by local ethics committees at sites where women were recruited. All approvals were granted prior study initiation. Participants signed a written informed consent before screening and enrolment. The trial was conducted under the Food and Drug Administration's Investigational New Drug application number 69,107.

Women were recruited on five different sites: a community clinic and a sexually transmitted infections clinic in Cotonou, Benin; the Y.R. Gaitonde Center for AIDS Research and Education (Y.R.G. CARE) in Chennai, India; the Medical Research Council in Durban, South Africa; Mulago Hospital (Makerere University) in Kampala, Uganda; and in clinics in Mudhol and Jhamkandi in Karnataka, India (a collaboration with the Karnataka Health Promotion Trust, Bangalore).

Laboratory testing was conducted in one laboratory in Kampala, in two laboratories in Durban and Chennai and in three laboratories in Cotonou and Mudhol.

For sites using multiple laboratories, one of them was identified as the local central laboratory where more sophisticated tests such as amplification assays were performed. All study sites had at least one small on-site laboratory where HIV rapid tests were performed.

### Role of ICRL

The ICRL gave advice on the selection of local laboratories, on laboratory algorithms, and on the choice of tests to be used for the study. It provided local laboratories with standardised testing methods for the investigation of end-point results and supported them in the redaction of SOPs. The ICRL was responsible for the laboratory trainings, including state of the art hands-on training at study initiation in each site, provision of control panels and repeated testing of a subset of specimens for quality assessment purposes. External clinical monitors also evaluated some laboratory aspects at each of their monthly visits. The ICRL performed an annual supervision visit for each laboratory and participated in the close-out visits. The first visit took place within 6 months of study initiation.

### Selection of the NG/CT amplification assay

Reliability of test results starts with the suitability of the selected test method or technique. The investigators and ICRL decided to use a molecular amplification assay for the determination of genital gonococcal and chlamydial infections. All local laboratories providing *N. gonorrhoeae* (NG) and *C. trachomatis* (CT) results had to use the same technique and testing method. In order to assess the interference of the CS microbicide gel on the different amplification assays, an *in vitro* and *in vivo* study was conducted at the ICRL. This assessment showed that the Amplicor CT/NG PCR (Roche, Molecular Systems, Branchburg, NJ) was inhibited by the CS gel. The SDA BD ProbeTec ET CT/NG (Becton Dickinson, Sparks, MD) assay was not affected by the CS gel or placebo, and was therefore selected for the clinical trial [2].

### Training of the laboratories at the study sites

At each study site, the international clinical study team provided training six weeks before study initiation on the clinical study protocol. A staff member from the ICRL joined the international clinical study team and conducted a separate training for all laboratory staff. The laboratory training focused on SOPs of the pre-analytical, the analytical and the post-analytical phase of the study. It included: group reading and discussion of the SOPs; problem solving exercises through case presentations; exercises with worksheets and other study forms.

Special attention was given to the additional instructions provided to guarantee the transparency, accuracy and reliability of the laboratory results. At the end of the training a wrap-up session with both clinic and laboratory teams was held to fine tune the final decisions regarding the pre-and post-analytical procedures.

Hands-on training for the selected molecular amplification assay was organized at the ICRL premises. Shortly after or at study initiation a follow-up training in finger-prick collection, HIV rapid testing and molecular amplification assay was conducted at the study site itself. Due to concerns related to safety and occupational exposure to HIV, it was decided that the finger-prick collection had to be performed with a Glucolet 2 automatic lancing device (Bayer HealthCare LLC, Mishawaka,USA). None of the study sites had any experience with this device. Although all study sites had experience with the HIV rapid testing, the ICRL re-trained them for reasons of standardization.

The staff member from the ICRL also provided guidance to the external clinical monitors regarding monitoring of specific laboratory-related issues.

### Quality control activities

#### HIV rapid tests lot validation

All tests and reagents were bought locally by the study sites. Verification of the new batches of HIV rapid tests and reagents was ensured by testing a batch validation panel prepared by the ICRL. The ICRL had to approve the results obtained with validation panels before any new HIV test batch could be used. The batch validation panels were manufactured in the same way as the quality control panels. Manufacturing of the panels is discussed hereafter.

#### Quality control panels

Every two months the study site laboratories were asked to test a quality control (QC) panel assembled by the ICRL, and which was identical for the different study sites. Panels were provided for HIV rapid testing and for CT and NG molecular amplification assay, respectively. Per test parameter, panels consisted of the same specimens ordered differently. For example: specimen labelled with number 1 in panel A for HIV testing, was labelled with number 2 in panel B and so on. A back-up from each study site panel was kept and tested at the ICRL. The study sites tested the first panel just before or at study initiation.

**Figure 1:**
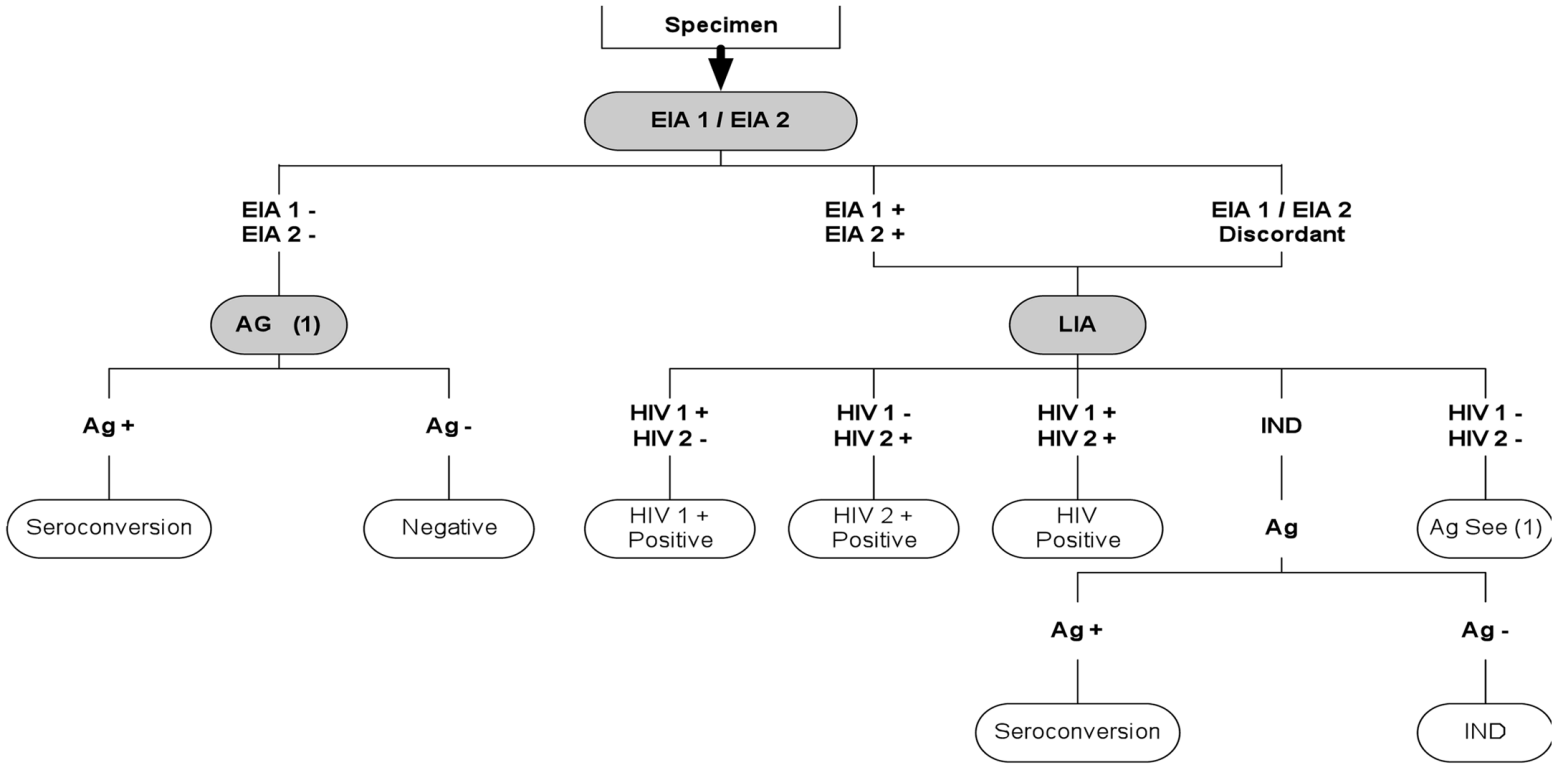
HIV testing algorithm used for quality control and confirmation. EIA 1: Enzygnost® Anti-HIV 1/2 Plus, EIA 2: Vironostika® HIV Uni-Form II *plus O*, LIA: INNO-LIA™ HIV I/II Score. Ag: INNOTEST® HIV Antigen mAb, IND: indeterminate.

The panels for HIV quality control and HIV batch validation consisted of 3 (2 negative and 1 HIV-positive) and 5 (3 negative and 2 HIV- positive) specimens, respectively. The HIV negative specimen was commercially purchased from PAA laboratories, Pashing, Austria. It consisted of human serum type AB from donors without AB antibodies. The positive specimens were selected from the ICRL's plasma collection. The collection consists of leftover specimens previously tested for HIV diagnosis, made anonymous and archived for future use in evaluation studies or for inclusion in quality control panels. Specimens included in the panels were tested at the IRCL using the HIV testing algorithm presented in Figure 1. In addition the specimens were also tested with the rapid tests used in the clinical trial: Determine™ HIV1/2, Uni-Gold™ HIV, and SD Bioline HIV ½ 3.0. All HIV positive specimens were inactivated at 56°C for 30 minutes.

Immediate action would be taken in case any of the study sites laboratories obtained HIV testing results different from those obtained at the ICRL.

The panels for CT and NG amplification testing were prepared by diluting a known concentration of elementary bodies (EBs) of CT and a known concentration of colony forming units (CFUs) of NG in diluted Phosphate Buffered Saline (pH 7.4) (1 part of PBS and 9 parts of saline).

The EBs of CT were obtained after culture of a *Chlamydia trachomatis* L2 strain onto a monolayer of McCoy cells with Complete Medium with Antibiotics (CMA). After 7 days of incubation at 35°C with 5% CO_2_, the culture was transferred in 50 mL conical tubes and centrifuged for 3 min at 1000 rpm. The EBs remain in suspension and the supernatant was filtered through a 0.8 µm filter. The EBs migrate through the filter, and the concentration of the EBs was determined by counting the EBs in tenfold dilutions of the filtered CMA suspension in diluted PBS. Counting was performed using an immunofluorescence colouring test, Micro Trak *C. trachomatis* Culture Confirmation Reagent (Trinity Biotech Plc, Wicklow, Ireland). Finally, 2 dilutions in PBS (1∶9, PBS:saline) containing each 3.10^6^ EBs/ml and 3.10^3^ EBs/ml were included in the QC panels.

The CFUs of NG were obtained after culture of a NG ATCC 49226 strain on blood agar. A suspension of 0.5 McFarland in diluted PBS was prepared from an overnight culture at 35°C and 5% CO_2_. Subsequently 10 fold dilutions were prepared from the 0.5 McFarland suspensions and from each dilution.25 µl were spread on the surface of a blood agar with IsoVitalex (Becton-Dickinson, Sparks, MD). Colonies were counted after an overnight incubation at 35°C and 5% CO_2_. Dilutions of 10^7^ and 10^4^ were selected for inclusion in the QC panel. One specimen from the QC panel was prepared by mixing dilutions containing 3.10^4^ EBs/ml and 10^5^ CFUs/ml. The negative specimen consisted of molecular biology grade water.

The QC panels for CT/GC contained 6 specimens of 250 µL each. Specimens were tested at the ICRL with the SDA BD ProbeTec ET CT/NG (Becton Dickinson, Sparks, MD) and the Amplicor CT/NG PCR (Roche, Molecular Systems, Branchburg, NJ) before the panels were assembled and shipped.

The study sites laboratories were allowed to miss detection of the low concentrated specimens. However the results of the strong positive and negative specimens were required to be accurate, if not, the IRCL would contact the study site and investigate the possible cause of discordant results.

#### Re-testing of specimens at ICRL

Per study protocol, the study sites had to collect two endocervical swab specimens. One swab was tested locally; the second was stored at −20°C for future quality control testing at the ICRL. The local laboratories shipped all specimens positive for NG and/or CT and every tenth specimen which tested negative for both microorganisms. The swabs were shipped every 6 months to the ICRL, The ICRL allowed a maximum of 5% of results obtained at the study site to be discordant. Percentages of discordant results exceeding this criteria, resulted in an alert signal given to the study site laboratory, which had to stop the testing and take corrective actions. The results obtained at the study site had to be invalidated in case a rate of more than 15% discordance was observed.

The study sites tested serum at screening and plasma at enrolment according to their respective national HIV testing algorithms. A serum aliquot of all specimens tested positive for HIV antibodies and 10% (every tenth) of the specimens which tested negative at screening, were shipped to the ICRL every 6 months. For follow-up visits, study sites had to use a study specific HIV testing algorithm on finger-prick blood. A plasma specimen was collected from participants who tested positive for HIV antibodies on the finger-prick specimen, and this was shipped to the ICRL. All shipped specimens were re-tested for quality control purposes and validation of results using the ICRL HIV testing algorithm presented in Figure 1.

Reports of the results of external quality control panels testing and of the re-testing were communicated to local laboratories. When necessary, advice on how to improve the performance was provided.

### Shipments and transports

Commercial couriers were contracted for the overseas shipment of study specimens and for the shipment of the QC panels from the ICRL to the study sites.

In Benin it was not possible to contract a courier and shipment by cargo was organised by the ICRL. The specimens were shipped on dry ice and according to the International Air Transport Association (IATA) regulations. Transport of the specimens within a study site or country was under the responsibility of the study site. For specimens that had to be kept cool or frozen, cool boxes with ice packs or dry ice were used, respectively.

All shipments and transports were accompanied with lists summarising the specimens included. The chain of custody was applied as described in the study specific SOPs.

### Monitoring and supervision visits

To assess day to day quality of HIV testing at study sites, the external clinical monitors registered on a monthly basis the number of positive test results obtained with each of the HIV rapid tests. The study specific HIV testing algorithm consisted of serial testing using a maximum of three rapid tests. The first rapid test used was the Determine™ HIV1/2 test, if this test gave a reactive result the SD Bioline HIV ½ 3.0 rapid test was performed, if this was not reactive, the Uni-Gold™ HIV test was used. A final HIV result was positive based on two reactive HIV rapid tests. Discordance of test results between the first and second test and between second and third test had to be below 5% [3].

A member of the ICRL visited the laboratories once a year, or following special request by the study principal investigator. During this supervision visit the raw data were examined, equipment maintenance was checked, competence of the laboratory staff was assessed, the stock of reagents and consumables was looked at and if necessary a hands-on training was provided. Advice and corrective actions were formulated in a visit report.

**Figure 2:**
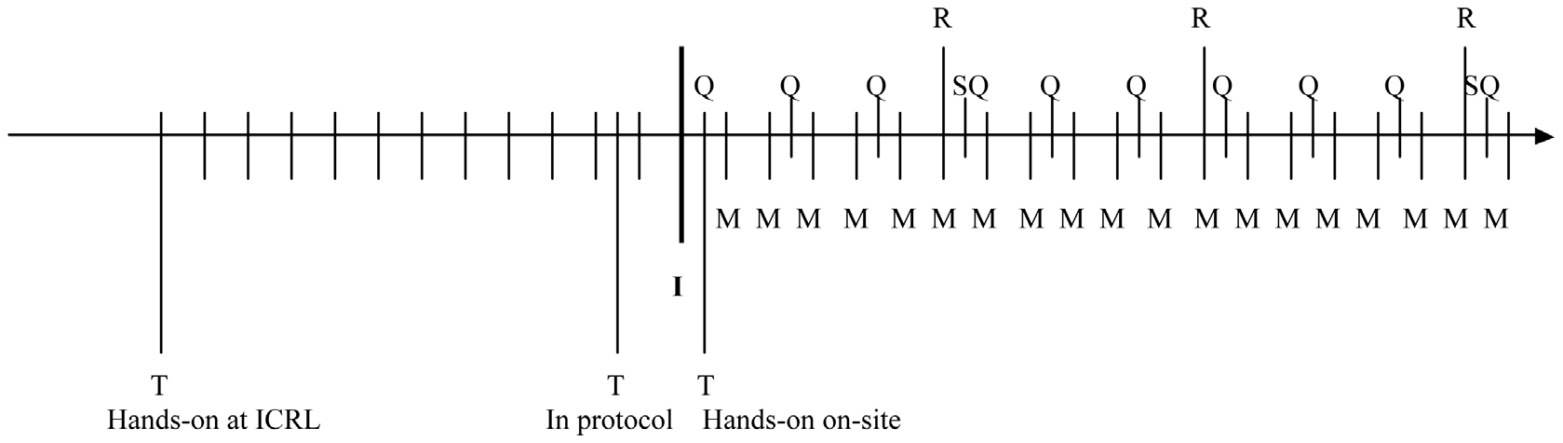
Timeline of quality assurance activities. T: lab training, I: study initiation, M: monthly monitoring visits, Q: testing of quality control panels, S: supervision visit, R: shipment of specimens to ICRL for re-testing.

Figure 2 presents the timeline of the different quality assurance/quality control activities which are also summarized in Table 1.

**Table 1:** Overview of quality assurance/quality control activities.

Type of activity	In charge	Details	Schedule
Supervision and training	ICRL	Hands on training in MAA (CT/NG SDA) at ICRL	Before study initiation
		Site training in study protocol (focus on pre-analytical, analytical and post-analytical procedures)	6 weeks before study initiation
		Hands on onsite training in sample collection and processing	2 weeks after study initiation
		Hands on onsite training in MAA (CT/NG SDA)	2 weeks after study initiation
		Supervision visit	Annually and if request of site PI
Monitoring	External study monitors	Review of diagnostic tests results and lab specific aspects (under ICRL guidance)	Monthly
Quality control for diagnostic tests	ICRL	Onsite testing of HIV and MAA QC panels	At initiation and every 2 months
		Re-testing for all positive and 10% negative specimens by HIV testing (serum)	Every 6 months
		Re-testing for all positive and 10% negative specimens by MAA testing (endo-cervical swabs)	Every 6 months
		Re-testing reports communicated to local sites	2–3 months after dispatch of specimens to ICRL

MAA: Molecular amplification assay.

## Results

The laboratory supervisor or manager from each study site was invited for a hands-on training in the SDA BD ProbeTec assay at the ICRL. At the end of the two-week training period, trainees received a panel of specimens to analyze. They were declared competent if they obtained 100% correct results for the analyzed panel and performed the testing according to the study SOP.

In order to communicate immediately HIV test results to enrolled study participants, it was decided to perform the HIV testing at the study clinic, using finger-prick blood and HIV rapid tests. If the finger-prick blood was HIV-antibody positive, a second specimen was collected and re-tested with the same HIV testing algorithm. The participant was informed about her serostatus only after confirmatory testing of the second specimen. Collection and testing of this second distinct specimen was requested to control for clerical errors or specimen mix-up. During follow-up, a total of 46/5734 specimens were initially HIV antibody positive according to the rapid testing algorithm; however 4/46 of the second collected specimens tested negative. The ICRL was at each occasion informed and consulted. Additional testing and investigation by the study sites, the international investigators and ICRL could confirm that the results of the first collected specimens were false positive reactions, and that no mixing up of specimens or clerical error had occurred. Two false positive HIV results on the first specimen were obtained in the same study site shortly after study initiation and within a short period. In order to advise and guide the local laboratory staff in charge of testing, a member of the ICRL went to the study site to examine the cause of the false positive results. The ICRL also observed that the discordance between the reactive HIV results obtained with the Determine test and SD Bioline exceeded 5% in this site. One of the findings was that the Determine test strips, once in use, were not kept according to the manufacturer's recommendation, i.e., in a sealed pouch with desiccant. The strips were kept in the refrigerator. The humidity of the refrigerator, but also of the room atmosphere, could have deteriorated the testing device especially if left on the bench during the day and put back in the refrigerator without adding a desiccant. Another finding was that the results were read as reactive by the laboratory technicians, even when only a very faint line was visible. The laboratory staff was re-trained on the appropriate storage of the strips and on the HIV rapid testing procedure with special emphasis on reading and interpretation. Information and additional instructions were also provided to the other study sites in a preventive manner.

All new batches of HIV rapid tests and of SDA reagent passed the lot control.

Excellent results were obtained for the quality control panels for HIV testing, as no false results were reported by the study sites. Among the five study sites a total of 168 control specimens, distributed in 28 panels, were tested for CT and NG. False negative results for CT and/or NG were obtained in each site. Four and nine false negative results were reported for CT and NG, respectively. The false negative results were obtained for specimens with a lower concentration of organisms. Of the four specimens which were reported negative for CT, 3 contained 3.10 ^3^ EB/ml and one 10^4^ EB/ml. Of the nine specimens which were reported negative for NG, 6 contained 10^4^ CFU/ml and 3 contained 10^5^ CFU/ml. None of the false negative results was caused by inhibition. All laboratories were advised to take more care while pipetting.

To assess the quality of the HIV serology testing, a total of 1238 out of 2916 specimens collected at screening were re-tested at the ICRL. Through the re-testing of these specimens for HIV, the ICRL detected in one particular site a problem of mislabelling of specimen aliquots before storage, and of clerical errors on the shipment list. The ICRL investigated with the concerned study site the possible causes of these errors. Fortunately, as the mislabelling and clerical errors occurred after the HIV rapid testing and delivery of result to the participants, no wrong results had been communicated to study participants. For all other study sites the results were concordant.

The ICRL confirmed all HIV seroconversions detected during the study using the testing algorithm presented under Figure 1.

The re-testing of endocervical specimens for CT and NG at the ICRL showed that some sites faced a problem of DNA contamination. To avoid further contamination and/or new contamination problems, all study laboratories were instructed to perform an environmental DNA check every two months. CT and/or NG DNA contamination was detected in all study sites laboratories performing the SDA test. The contaminated areas included the amplification area, air conditioning, keyboard and screen of the ProbeTec instrument, door handles, work bench, and fridge. During the period of DNA contamination the testing was put on hold, and resumed after thorough cleaning with bleach and only after approval by the ICRL. Aside from the regular environment checks, the laboratories were asked to do an extra cleaning when more than 5% false positive results were detected for either CT or NG following re-testing at the ICRL. This criterion was exceeded twice by the same site, once for the CT detection and once for the NG detection, another study site exceeded once the criteria for the CT detection. Positive results were considered to be false positive if the on-site positive amplification results could not be confirmed by the ICRL. False negative results for CT and NG was observed once in 1 study site and false negative results for NG was observed twice in another site, and represented less than 1% of the re-tested specimens. The women, who were not treated due to a false negative result, were treated at the next occasion following notification of the false negative result. Table 2 presents the numbers and percentages of CT and NG false results obtained in the study sites.

**Table 2:** *Chlamydia trachomatis* and *Neisseria gonorrhoeae* amplification re-testing results.

Site	Number of specimens	CT + ive results %	GC+ ive results %	CT false +ive results % (N)	GC false +ive results % (N)	CT false –ive results % (N)	GC false –ive results % (N)	Total false results % (N)
A	120	25.0 (30)	32.5 (39)	5.8 (7)	1.7 (2)	0	0.8 (1)	8.3 (8)
	125	38.4 (48)	27.2 (34)	0.8 (1)	0.8 (1)	0	0.8 (1)	2.4 (3)
	145	29.0 (42)	25.5 (37)	4.8 (7)	2.8 (4)	0	0	7.6 (11)
	39	17.9 (7)	35.9 (14)	0	2.6 (1)	0	0	2.6 (1)
B	108	22.2 (24)	46.3 (50)	0.9 (1)	5.6 (6)	0	0	6.5 (7)
	100	14.0 (14)	30.0 (30)	6.0 (6)	3.0 (3)	0	0	9.0 (9)
	48	8.3 (4)	27.1 (13)	0	2.0 (1)	0	0	2.0 (1)
C	146	34.9 (51)	27.4 (40)	4.1 (6)	4.1 (6)	0.7 (1)	0.7 (1)	9.6 (14)
	72	25.0 (18)	16.7 (12)	4.2 (3)	2.8 (2)	0	0	7.0 (5)
	208	23.1 (48)	14.9 (31)	1.9 (4)	0.5 (1)	0	0	2.4 (5)
	32	18.8 (6)	12.5 (4)	0	3.1 (1)	0	0	3.1 (1)
D	137	9.5 (13)	0	0	0	0	0	0
E	10	10.0 (1)	10.0 (1)	0	0	0	0	0

CT: *Chlamydia trachomatis*, NG: *Neisseria gonorrhoeae*, +ive: positive; -ive: negative, N: number.

## Discussion

The local laboratories in the CS multi-country study performed well. All HIV seroconversions detected at the study sites were confirmed by the ICRL, the results of the HIV control panels were correct for all sites, no discordant results were obtained in the re-testing of a sub-sample of specimens collected at screening for HIV, except for one site, and whenever CT and/or GC DNA contamination was detected, testing only resumed after confirmed decontamination.

Recently a quality management system for laboratories collaborating in clinical trials was proposed by the British Association of Research Quality Assurance, and translated into Good Clinical Laboratory Practices guidelines [4]. The National Institute of Allergy and Infectious Diseases also published their guidelines on GCLP and currently further works aiming at the harmonization of different GCLP guidelines are ongoing [5], [6]. Overall, the GCLP guidelines provide a framework on how to deliver high quality, scientifically valid clinical trial laboratory data. However as illustrated by Wertheim et al, challenges exist for the implementation of these guidelines in resource constrained settings [7]. They must be implemented in such a way that they are in line with the realities of the setting and respond to the local needs. Local laboratories may wish to acquire new techniques rather than ship the specimens overseas for testing. In complement to the procurement of new equipment and reagents, laboratory staff needs to be trained in the maintenance of equipment, procedure of the new technique and in assay validation. It has to be noted that in contrast to the resource rich settings, resource constrained settings lack logistic and technical support from manufacturers and providers. Very often support provided by the manufacturer remains restricted to the installation of the equipment and demonstration of the technique. Resource rich settings have more readily access to maintenance contracts, easy communication, and are provided with continuous training under the form of symposia organized by manufacturers. Therefore close supervision is recommended at the installation of a new technique in resource constrained settings, and once the staff is accustomed to the technique, guidance can then be slowly phased out [8], [9].

The CS trial has distinguished itself by the special emphasis put on laboratory activities via a tailor made approach to capacity building, monitoring and supervision of technical staff both at initiation and throughout the study. This aspect is indeed not routinely included or required in the set-up of clinical trials taking place in resource rich settings, where sponsors will select laboratories with pre-existing and well documented credentials based on international norms. A significant aspect of capacity building activities consisted in the provision of hands-on training in laboratory analysis and implementation of a proficiency testing program externally monitored by the ICRL, again not a frequent practice in clinical trials. The role of clinical research associates (CRAs) or study monitors is principally to train the study sites with regards to the study protocol, rather than to ensure the oversight of laboratory methods or to consider the implementation and management of quality systems in the laboratory. Accordingly, whereas source laboratory documents verification is routinely performed by monitors and CRAs, assessment of laboratory performance with regards to SOP compliance, or of effective management and implementation of quality systems, is often beyond the scope of the standard monitoring process. These aspects rather pertain to the laboratory audit, which provide a “snap shot” appraisal of performance, but which purpose is not to train on a continuous basis.

Another unique feature of the CS trial set up was the use of custom made quality control panels for external technical performance assessment. Although in-house quality control panels can only be used after thorough validation of the manufacturing process, advantages are many. Firstly, commercial quality control panels are not always available for all parameters to be analysed. Further, panel concentrations can be chosen in function of study-specific end point determinations and according to the sensitivity and specificity of laboratory methods used. Subscription to an external and independent proficiency testing scheme could be an option, but this would exclude the individual laboratory coaching process specific of the CS trial. Indeed proficiency testing providers typically give feedback on expected results to participating laboratories in the form of a detailed report, but they do not provide individual assistance for performance improvement if needed. It should also be noted that as participation to proficiency testing schemes is confidential, sharing of performance related information with study sponsors or third party is at the discretion of the laboratory.

What would have happened if a quality assurance program had not been included in the study specific laboratory activities? We can only speculate, but we do have indications that in one study site, aliquot mislabelling and clerical errors could possibly have affected the study data. The use of an amplification assay for the detection of CT and NG facilitated the study in terms of specimen collection and transport, but the laboratory sites faced problems with DNA contamination. If the contamination had not been detected, false positive results could have been obtained leading to over-treatment of study participants. Apart from the four HIV false positive results on the first collected specimen and the more than normal discordance rate between results of first and second or third rapid HIV tests seen in one study site at the beginning of the study, and which was readily corrected, we did not detect problems with the HIV testing.

In summary, we were able to guarantee and to assure that laboratory data for the endpoint determinations as stipulated by the study protocol were reliable and of high quality. The efforts put in capacity building of the study laboratories were very fruitful, as none of the testing had to be repeated in the ICRL to be included in the data base. The on site laboratories gained experience in the performance of testing and in the documentation required for phase III clinical trials. They acquired a new technique, i.e., SDA, which can be used in future clinical trials or for diagnosis if funding is available.

We also remarked that although the testing of panels for quality control purposes could give us some indications concerning the quality of the testing, it was through re-testing a sub-sample of the specimens and monitoring of the in country laboratory activities that potential major problems could be detected. Indeed we believe that participation in external quality control or proficiency testing (PT) schemes is not enough to measure the performance of a laboratory. The laboratory results of EQC or PT schemes reflects the potential analytical performance of a laboratory on the technique used, it does not reflect the pre-analytical and/or post-analytical performances.

While designing the quality assurance program we were vigilant to avoid overloading the local laboratory, to make it as easy as possible, feasible and most importantly acceptable by the local laboratory staff. We were very aware that clerical errors may be frequent in laboratories and we therefore tried to restrict the numbers of forms and sheets to fill in. We also wanted to be flexible so that existing activities regarding quality control and assurance in the local laboratory could be maintained. However the set-up of our quality assurance program showed some shortcomings. In our design of capacity building and quality assurance activities we focused on the laboratory tests needed for the endpoint determinations. We did not pay much attention to the tests performed in the context of additional diagnosis and treatment for other sexually transmitted or reproductive tract infections provided as a service to the study participants. Indeed, we observed for example that problems occurred with the diagnosis of bacterial vaginosis. Ad hoc on-site trainings were provided during supervision visits. Previously reported results were questioned and corrected if necessary, but for one site we were not able to correct the previous reported results because the biological material and hard copy raw data had been destroyed. Strikingly, this happened in an accredited laboratory, which sub-contracted the laboratory testing, and provided services to many clinical trials. This stresses the fact that working with accredited laboratories is not a guarantee of absolute quality and that those laboratories also need continuous guidance.

It is clear that site capacity building, in particular upgrading laboratory facilities, and setting up a quality assurance program in a clinical trial increases the overall study budget. These activities ended up representing approximating 10% of the total budget for this multi-center clinical trial but were expected to range from 5–10% if the trial had not been terminated early. However since most trial endpoints, including the primary endpoint in an HIV prevention trial, are laboratory based, this extra investment is worth the cost. The extra budget should include training of local laboratory staff in a central laboratory and on-site, the costs of the supervision visits, the extra time the laboratory staff spends in applying the quality assurance or quality systems, the additional testing ( for validation, quality control, batch validation, environmental checks, training) requested at the local laboratory as well as at the ICRL, the preparation and shipment of quality control and batch panels, the shipment of specimens for re-testing from the study site to the central laboratory, equipment maintenance and calibration contracts.

In conclusion we want to urge sponsors to include in their clinical trials a strong capacity building and upgrading component for the laboratories that support the trials. The full application of the GCLP guidelines in clinical trials needs appropriate resources, and we strongly believe that sponsors and donors should consider it essential in order to ensure the protection of participants and the reliability of final data. To date the application of the GCLP guidelines in clinical trials has been optional, but we think that it will become a mandatory and a necessary requirement in the near future. The transparency, traceability and validity of the laboratory data outweigh the extra laboratory activities and costs in clinical trials.

At the end the overall population served by the laboratory will benefit from the upgrade of the services [10], even outside the framework of clinical trials.
